# Nonprescription fish antibiotics: Are they the same as those prescribed to humans?

**DOI:** 10.1016/j.jfscie.2022.100015

**Published:** 2022-10-26

**Authors:** Eugenia Popescu Roberts, Charles Veltri, Maria Lozoya, Gina Agostini-Walesch, John C. Mitchell

**Affiliations:** a College of Dental Medicine, Midwestern University, Glendale, AZ; b Department of Pharmaceutical Sciences, College of Pharmacy, Midwestern University, Glendale, AZ

**Keywords:** Amoxicillin, cephalexin, drug abuse, drugs/pharmacology, self-medication

## Abstract

**Background:**

Patients at a dental school were observed to self-medicate for dental pain and presumed infection with nonprescribed ornamental fish antibiotics, thereby circumventing professional health care. This study determined if the human-approved antibiotics, amoxicillin and cephalexin, were present in the nonprescribed, over-the-counter fish antibiotics.

**Methods:**

Human-grade prescribed and over-the-counter commercially-available fish antimicrobials (amoxicillin, cephalexin) were analyzed by using high-performance liquid chromatography ultraviolet-visible detection following US Pharmacopeia (USP) protocols. The contents of 20 capsules of each type were combined and dissolved in a carrier fluid to a concentration of 1 mg/mL. Samples were analyzed using high-performance liquid chromatography at a flow rate of 1.5 mL/min using isocratic mobile phase conditions.

**Results:**

All products contained the equivalent of not less than 90.0% and not more than 120.0% of their labeled contents, within the USP standards. Although no major impurities were identified, there was evidence of several as-yet unidentified excipient ingredients. Results confirm that the human-grade prescribed and nonprescribed over-the-counter fish antibiotics tested match USP standards and are pharmacologically indistinguishable.

**Conclusions:**

The results of this study showed that the major component of the amoxicillin and cephalexin capsules marketed for fish contain their purported levels of antibiotics.

**Why Is This Important?**

Patients have access to over-the-counter small animal antibiotics online or at pet stores without a prescription owing to a loophole in veterinary prescription laws. The use of these unregulated products by humans may contribute to the growing rates of antibiotic resistance and associated economic and health care burdens.

## Introduction

For over 60 years, antibiotics have been invaluable for treating infections in human and veterinary medicine. However, the overuse and misuse of antibiotic agents by patients and prescribers not only decrease drug efficacy but also increases the likelihood of antibiotic resistance and allergic potential.^1^ Self-medication with antibiotics has been shown to contribute to antibiotic resistance^2^ and has become a global concern in different communities, populations, and countries.^3^, ^4^, ^5^ For these and other reasons, many domestic and international policies are in place to circumvent potential abuses by restricting antibiotic access by the general public and implementing audits and oversight of health care entities.^6^^,^^7^ In the United States, 2 regulatory agencies oversee antibiotic use in humans and in animals in veterinary or agricultural contexts: the US Food and Drug Administration (FDA) and the US Department of Agriculture. However, antibiotics marketed for ornamental fish (non–food-producing animals) do not fall under the purview of either entity, presenting a loophole that provides easy antibiotic access through brick-and-mortar pet stores and online vendors.^8^ As long as antimicrobial products are labeled as “Not for human consumption,” it is deemed legal to sell these medications over-the-counter. The online availability of nonprescribed antibiotics is not new but seems to be on the rise. Over a 90-day period in 2014, approximately 70,000 capsules of pet amoxicillin were sold on an online auction website.^9^ Although over-the-counter antibiotics marketed for pets are specifically labeled as not for human use, easy access may facilitate inappropriate dosing, distribution, and use of these drugs. The exponential growth of web-based purchasing and economic hardships that fuel medicine misuse^10^ likely means the rates of nonprescribed antibiotic use are increasing.^11^ This is likely exacerbated by the 2019 emergence of COVID-19, which has been associated with a marked increase in global antibiotic use,^12^, ^13^ some of which is due to self-medication outside of medical guidance.^14^

Self-medication in the United States with nonprescribed antibiotics has been reported to range from 1% through 66%.^5^ Instances of self-medication with fish antibiotics obtained from online sources have been reported in published professional statements for the last 20 years; however, no policies have been enacted to curtail this practice.^8^^,^^15^ A 2020 study by Zhang et al^11^ evaluated the content of text-based reviews and comments accompanying online fish antibiotic marketplaces and identified 55 statements consistent with intended human use. Although this represented a small portion of the overall data (< 3% of reviews posted), comments related to intended human use generated significantly more traffic than other reviews and comments, as indicated by the number of likes and dislikes the comments received.^11^

In 2019, several patients at a large dental school clinic reported having used several forms of fish antibiotics to self-treat dental pain and self-diagnosed infection. These antibiotics were marketed for use in ornamental fish and were purchased by the patients online or at local pet stores. This circumvention of professional health care was a cause for concern. Self-medication may increase the risk of drug resistance, interact with a patient’s prescribed medication regimen, cause adverse reactions, and delay the timeline for diagnosis and proper treatment.^16^ An additional risk relates to uncertainty; because antibiotics marketed for fish do not fall under any official regulatory mechanisms, there are no assurances about the quality, content, or amount of ingredients therein. This study aimed to determine whether the purported amounts of antibiotics were present in the several common forms of nonprescribed fish amoxicillin and cephalexin antibiotics and if they differed from human-prescribed antibiotics of equal dosage.

## Methods

**Figure 1:**
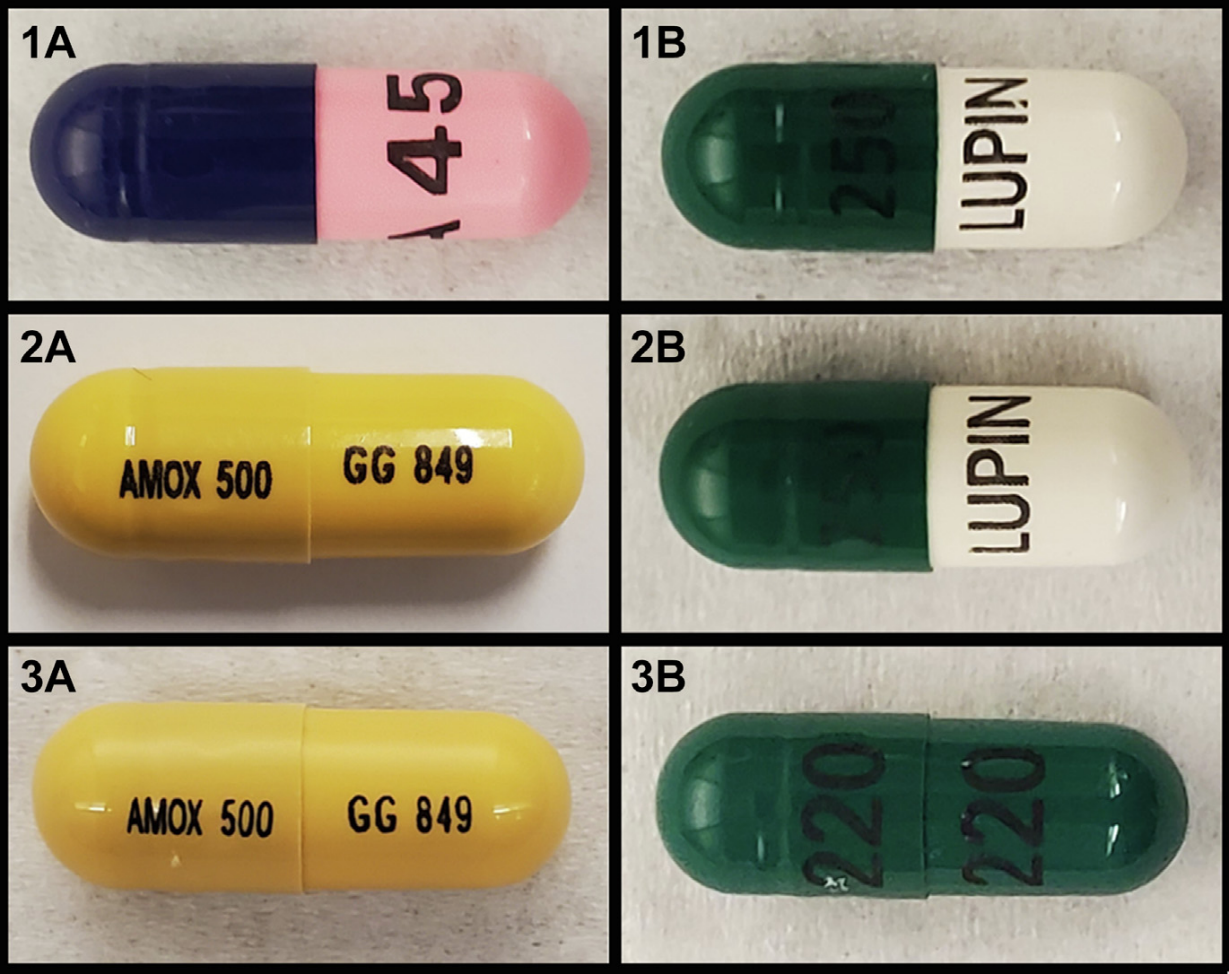
Photographic comparison of the 3 amoxicillin (left) and cephalexin (right) products tested: human-prescribed amoxicillin (**1A**) Fish Mox (Sandoz Pharmaceuticals) (**2A**), fish aid amoxicillin (**3A**), human-prescribed cephalexin (**1B**) Fish Flex (Lupin Pharmaceuticals) (**2B**), fish cephalexin (**3B**).

**Figure 2:**
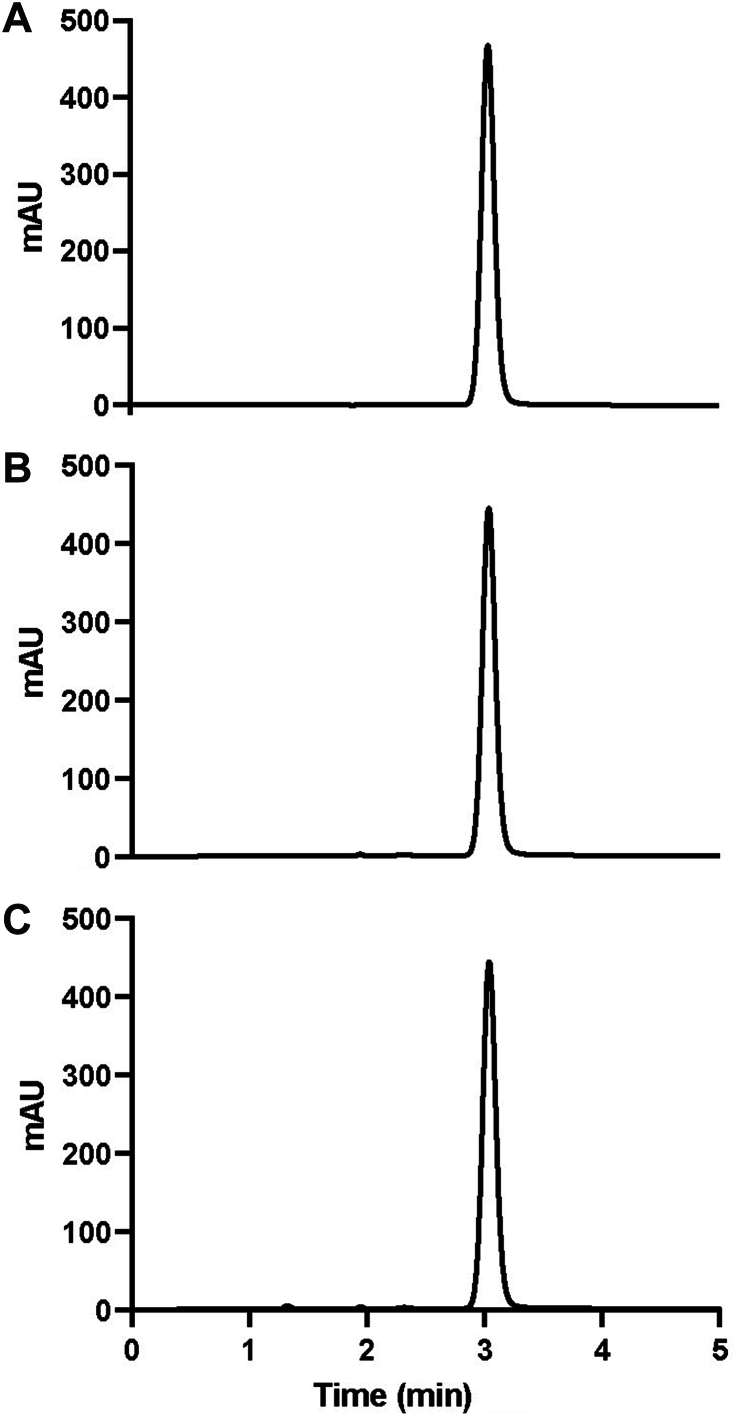
High-performance liquid chromatography chromatograms of amoxicillin commercially available samples. US Pharmacopeia grade standards (**A**) compared with Goldman Pharmaceutical Group **(B)** and Thomas Labs **(C)**.

Four commercially-available antibacterial fish medications (2 types each of 500 mg of amoxicillin, 250 mg of cephalexin) were purchased via an online pet vendor without a prescription. These came from two distributers. Those with the label names Fish Mox and Fish Flex were distributed by Thomas Labs, and those with the label names fish amoxicillin and fish cephalexin were distributed by Goldman Pharmaceutical Group under the brand name Fish Aid Antibiotics (Table 1). Comparable human-grade amoxicillin and cephalexin antibiotics were obtained by prescription at a local pharmacy chain (Table 2). Pharmacologic identifiers imprinted on each pill (Figure 1, Figure 2) were used to source each product’s labeler, supplier, National Drug Code number, and other relevant pharmaceutical information through online databases.

**Table 1:** US Pharmacopeia values and pharmaceutical information for each product.

Antibiotic Type	Product Name	Drug	Strength (mg)	Distributed by∗	Labeler or Supplier	National Drug Code No.	Percentage US Pharmacopeia Standard
Cephalexin	NA†	Cephalexin	250	NA	Lupin Pharmaceuticals	68180-0403	117.1
	Fish Flex	Cephalexin	250	Thomas Labs	Lupin Pharmaceuticals	68180-0403	108.3
	Fish cephalexin	Cephalexin monohydrate	250	Goldman Pharmaceutical Group	Ascend Laboratories	67877-0220	111.9
Amoxicillin	NA	Amoxicillin trihydrate	500	NA	Aurobindo Pharma	65862-0017	113.7
	Fish Mox	Amoxicillin trihydrate	500	Thomas Labs	Sandoz Pharmaceuticals	00781-2613	112.3
	Fish amoxicillin	Amoxicillin trihydrate	500	Goldman Pharmaceutical Group	Sandoz Pharmaceuticals	00781-2613	114.4

∗ As documented on the product label.

† NA: not applicable.

All antimicrobial agents were analyzed by means of high-performance liquid chromatography ultraviolet-visible detection following US Pharmacopeia (USP) monographs and protocols. A total of 20 capsules were opened and the contents combined. The powder was dissolved in a 50 mM potassium phosphate buffer (pH 5.0, amoxicillin) or deionized water (cephalexin) to a final concentration of 1 mg/mL. Samples were separated via liquid chromatography at a flow rate of 1.5 mL/min on C18 L1 columns using isocratic mobile phase conditions of acetonitrile to potassuim phosphate buffer is 1:24 and 5.6 mM sodium 1-pentasulfonate in a mixture with the ratio of acetonitrile to methanol to trimethylamine to water as 20:10:3:170, pH 3.0 for amoxicillin and cephalexin, respectively.

## Results

**Figure 3:**
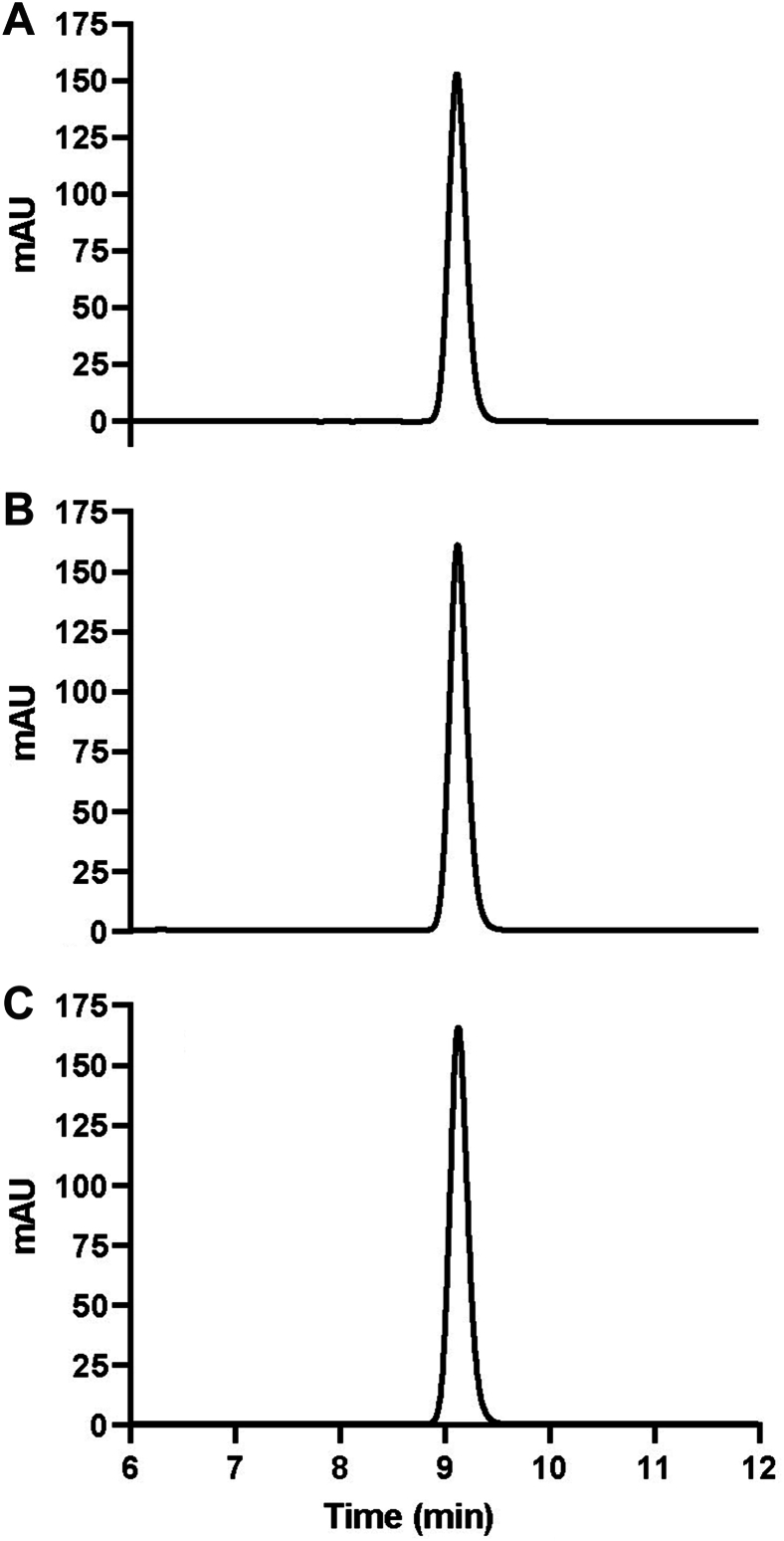
High-performance liquid chromatography chromatograms of cephalexin commercially available samples. US Pharmacopeia grade standards (**A**) compared with Goldman Pharmaceutical Group **(B)** and Thomas Labs **(C)**.

All 6 antibiotic products contained the equivalent of not less than 90.0% and not more than 120.0% of their labeled amount of amoxicillin or cephalexin (Table 1). No major impurities were identified in any of the products; yet, there was evidence of several as-yet unidentified excipient ingredients. Results confirmed that both human-grade prescribed and nonprescribed over-the-counter ornamental fish antibiotics we tested matched USP monogram standards and were pharmacologically indistinguishable (Figure 2, Figure 3).

**Table 2:** Description of the antibiotic products tested.

Antibiotic Type	Product Name	Strength (mg)	Intended Recipient	Prescription Required?∗	Purchasing Location	Pill Description	Pill Imprint
Cephalexin	NA†	250	Human	Yes	Local pharmacy	Green and white capsule	250 LUPIN
	Fish Flex (Lupin Pharmaceuticals)	250	Fish‡	No	Online pet retail vendor	Green and white capsule	250 LUPIN
	Fish cephalexin	250	Fish‡	No	Online pet retail vendor	Dark green capsule	220/220
Amoxicillin	NA	500	Human	Yes	Local pharmacy	Pink and purple capsule	A45
	Fish Mox (Sandoz Pharmaceuticals)	500	Fish‡	No	Online pet retail vendor	Dark yellow capsule	AMOX 500/GG 849
	Fish amoxicillin	500	Fish‡	No	Online pet retail vendor	Dark yellow capsule	AMOX 500/GG 849

∗ Refers only to whether a prescription was required by the authors to purchase the product.

† NA: not applicable.

‡ Specifically, fish not intended for human consumption, or aquarium and ornamental fish only.

Pharmacologic identifiers (Figure 1, Figure 2) on the capsules indicated that Thomas Labs sourced their cephalexin from the same supplier as the human cephalexin we tested. With regard to amoxicillin, Thomas Labs and Goldman Pharmaceuticals Group both sourced their product from the same supplier (Table 2).

## Discussion

Antibiotic stewardship in the dental profession is in center stage; 2019 recommendations from the American Dental Association encourage appropriate dental treatment and limit antibiotic use for dental pain.^17^ However, these continued professional efforts may be circumvented by patients self-medicating with nonprescribed antibiotics. The results of our study showed that the primary ingredient in each antibiotic sample was, in fact, amoxicillin or cephalexin, as reported on the product label for both nonprescribed fish and prescribed human antibiotics. These results corroborate those of Zhang et al,^11^ who found the physical properties and pill imprints of 8 different types of fish antibiotics to be consistent with those marketed for humans, including the 4 brands evaluated here. However, these results differ from those of Farah et al,^18^ who found inconsistent amounts of ciprofloxacin in fish antibiotics sold via online vendors, with several capsules failing to meet the content standard. However, they corroborate reports by Egelund and Casapao,^15^ who found that “the majority of antibiotic dosages for fish are identical to human dosing—equivalent in active ingredients and dosage forms.” Our results add to those of these studies by showing that the chemical compositions of fish and human antibiotics are equivalent in dose and composition. These studies confirm that a broad range of antibiotics used for treating medical and dental illnesses are also available for ornamental fish, including amoxicillins and cephalexins. The FDA regulatory loophole concerning fish antibiotics gives the public easy, inexpensive access to prescription-quality antibiotics with no medical oversight.

These results are concerning because, as shown in a prior case report,^19^ nonregulated pet antibiotics are being used to self-medicate for presumed symptoms, including dental pain, without consulting health care professionals. As part of antibiotic stewardship, all levels of health care practitioners and pharmacists are encouraged to specifically include nonprescribed antibiotic use in medical history questionnaires so that potential treatment interactions can be avoided.^15^^,^^19^

A broad range of antibiotics is medically important in dentistry and medicine, including amoxicillins, penicillins, tetracyclines, and cephalexins, which are also available for ornamental fish. The FDA regulatory loophole concerning these small animal antibiotics gives the public availability without a prescription at local pet stores and from multiple online vendors. Fish antibiotics are intended to be in capsule or powder form to treat the infected water. Human antibiotics can be administered in tablet or capsule form and are absorbed in the gut. As these antibiotics, intended for ornamental pet fish, are manufactured without FDA or US Department of Agriculture oversight, they may contain chemical or microbe contaminates or be exposed to storage conditions that reduce the drug’s effectiveness and make it unsafe for human consumption. The drugs sold online may also be human-grade antibiotics but advertised and sold as fish antibiotics to get around FDA prescription guidelines.^20^

The misconception by the public regarding the safety and applicability of antimicrobial medications is of even greater concern. Patients often self-treat or request antibiotics for symptoms of a viral nature for which antimicrobials are ineffective.^16^^,^^19^^,^^21^ It is important to assess, with further study, the extent to which antibiotics not intended for humans (and any excipient ingredients therein) may affect long-term patient health and treatment outcomes. Anecdotally, patients who admitted to using nonprescribed antibiotics to treat symptoms associated with sore throat or dental infection, also had confusing diagnostic issues (eg, no radiographic lesions despite complaints of pain, limited benefit in pain relief, and complaints of generalized oral pain).^19^ This is presumably because of improper use and dosages of nonprescribed antibiotics to suppress chronic dental infections. Assessing the extent to which these symptoms were due to antibiotic self-medication and overuse is an important area of future research.

Further study is needed to understand the full scope and consequences of human consumption of over-the-counter non–FDA-regulated animal antibiotics.^22^^,^^23^ The director of the United Nations Interagency Coordination Group on Antimicrobial Resistance has called antibiotic drug resistance a silent tsunami and cautioned that current political momentum is insufficient.^24^ Spurred on by social media and internet forums, the public’s improper dosing, self-diagnosing, and easy circumvention of professional care because of access to over-the-counter antibiotics may also add a layer to antibiotic resistance.^15^ By 2050, the projected number of deaths from drug-resistant infections worldwide is expected to exceed 10 million people.^24^ Furthermore, deaths from drug-resistant infections may outnumber the death due to cancer, diabetes, and even traffic accidents combined.^25^ In the United States, the 2019 Centers for Disease Control and Prevention Antibiotic Resistant Threats report presents data showing that antibiotic-resistant strains of bacteria were responsible for 2.8 million infections and 35,000 deaths, and health care costs associated with treating antimicrobial resistance were reported to be roughly $20 billion annually.^10^^,^^26^ This number does not take into account personal out-of-pocket costs, loss of work and production, and broader economic ramifications therein.^27^

## Conclusions

The overuse and misuse of antibiotics is a shared concern among modern health care practitioners because of the increased prevalence of antibiotic-resistant bacterial strains and potential drug allergies or drug interactions. Although antibiotic overuse and nonadherence problems are well documented, less is known about the self-use of drugs labeled for small, non–food-producing animals, particularly ornamental fish. This easy access and circumvention of professional health care oversight is concerning. As verified by this study, the antimicrobials tested were pharmacologically indistinguishable from USP standards and one another; therefore, pet antibiotic use in humans should be considered by the health care community as an important avenue for nonprescription self-medication. In addition, this unregulated use may be a public health hazard adding to the antibiotic crisis of drug resistance and drug allergy potential.
